# Pedicle flap reconstruction for treatment of infected median sternotomy wounds after cardiac surgery in overweight and obese patients: proposal of a management algorithm based on a case series analysis

**DOI:** 10.1186/s12893-021-01451-5

**Published:** 2022-01-08

**Authors:** Marios Papadakis, Afshin Rahmanian-Schwarz

**Affiliations:** 1 Department of Surgery II, University Witten/Herdecke, Heusnerstr. 40, 42283 Wuppertal, Germany; 2 Department of Plastic, Reconstructive, Aesthetic and Hand Surgery, Klinikverbund Südostbayern AG, Traunstein Hospital, Wuppertal, Germany

**Keywords:** Sternum infection, Osteomyelitis, Obesity, Pedicle flap reconstruction, Algorithm

## Abstract

**Background:**

A relationship between obesity and adverse outcomes in patients with post-sternotomy wounds undergoing pedicle flap reconstruction is not well-documented. In this study, we present a single-centre retrospective case series analysis of early postoperative outcomes of patients with infected post-sternotomy wounds undergoing pedicle flap reconstruction. We also propose a management algorithm for such patients, based on BMI and wound width.

**Methods:**

We retrospectively analyzed all patients, who underwent pedicle flap reconstruction for major sternal wound infections after sternotomy for cardiac surgery in a tertiary hospital in Germany during a 5-year period. Exclusion criteria included patients younger than 18 years of age and patients with BMI < 18.5 kg/m^2^. Patients were divided into 2 groups according to BMI: normal-weight (NW; BMI < 25 kg/m^2^) and overweight/obese (OB/OW; BMI > 25 kg/m^2^). Both groups were compared in terms of preoperative parameters and early postoperative outcomes. Preoperative parameters included demographics, wound bacteria and comorbidities. Postoperative outcomes included duration of surgery time (from incision to skin closure), transfusion requirement (during surgery and entire hospital stay), onset of flap and donor-site complications, length of stay and 30-day mortality. We employed the two-tailed t-test to compare continuous variables and the two-sided Fischer’s exact test to compare categorical variables. Statistical significance was set at p < 0.05.

**Results:**

The total sample consisted of 48 patients. Overall mean BMI was 28.4 (6.1) kg/m^2^. Mean age was 67 (12) years. The study group consisted of 28 patients with BMI > 25 kg/m^2^, who were compared with 20 normal-weight patients. There was a significant difference amongst both groups regarding duration of surgery (120 vs. 174 min, p < 0.05). Donor-site complications requiring intervention were observed in 30% of patients in both groups. Flap-related complications were recorded in 16 (57%) cases in the study group and 7 cases in the control group (35%, p = 0.15).

**Conclusions:**

We conclude that wound width and BMI can aid the decision-making process for patients with infected sternal wounds after cardiac surgery requiring pedicle flap reconstruction. However, in our case series analysis, OB/OW patients were not found to be at statistically significantly increased risk for worse postoperative outcomes, but were associated with a longer duration of surgery.

## Background

Median sternotomy is the most common method to access the anterior mediastinum for surgical treatment of coronary heart disease. Despite being a generally safe method, infection complicates 0.5–5% of sternotomy wounds. Mortality rates from these complications are reported to range from 7 to 80% [1]. Sternotomy-related complications most often affect the presternal and sternal compartment and include wound dehiscence, abscess formation or underlying osteomyelitis. Most of these wounds are primary treated from cardiac surgeons. However, some patients do not respond and develop large defects and chronic wounds, requiring flap reconstruction and therefore are referred to plastic surgeons. Several musculocutaneous and muscle pedicle flaps have been utilized for such defects, the most pronounced being the latissimus dorsi flap, the pectoralis flap and the rectus abdominis flap. The omental flap is considered a second-line flap, reserved for selected cases where the regional flap options are either not available or have failed.

The effect of obesity on outcomes of breast reconstruction has been well-documented. Lee et al. report that obesity increases the risk of both flap-related and donor-site complications in free flap breast reconstruction [2]. Several studies have established a strong association between obesity and adverse outcomes in pedicle flap breast reconstruction and postbariatric surgery as well [3–6], whereas others could not find significant differences in complication rates between overweight/obese and normal weight patients [7].

However, a relationship between obesity and adverse outcomes in patients with post-sternotomy wounds undergoing pedicle flap reconstruction is not well-documented. Moreover, existing algorithms focus only on defect-related factors and do not take into account patient-related parameters that may affect the outcome [8]. Understanding the effect of obesity on early outcomes after pedicle flap reconstruction for post-sternotomy wounds may improve preoperative risk stratification, facilitate patient counselling and aid in decision-making for surgical approach optimization. In this study, we present a single-centre retrospective case series analysis of early postoperative outcomes of patients with infected post-sternotomy wounds undergoing pedicle flap reconstruction. This is also the first study to propose a management algorithm for such patients, based on BMI and wound width.

## Methods

### Patients demographics

Following institutional review board approval, we retrospectively reviewed and analyzed the computerized medical records of all patients, who underwent pedicle flap reconstruction for major sternal wound infections after sternotomy for cardiac surgery adenoma in a tertiary hospital in Germany during a 5-year period. Exclusion criteria included patients younger than 18 years of age and patients with severe malnutrition, i.e. patients with BMI < 18.5 kg/m^2^. Patients were divided into 2 groups according to BMI: normal-weight (NW; BMI < 25 kg/m^2^) and overweight/obese (OW/OB, BMI > 25 kg/m^2^). Both groups were compared in terms of preoperative parameters and early postoperative outcomes. Preoperative parameters assessed, included demographics (gender, age), wound bacteria and comorbidities. Physical status and overall perioperative risk was classified according to the American Society of Anesthesiologists’ physical status classification system (ASA). ASA score is a good and reliable indicator of comorbidity, but in our case all patients underwent sternotomy for cardiac surgery, which means an ASA score of ≥ 3. For this reason, it was categorized as III or less, or IV or greater. Postoperative outcomes included duration of surgery time (from incision to skin closure), transfusion requirement (during surgery and entire hospital stay), onset of flap and donor-site complications, length of stay (LOS) and 30-day mortality. Complications are defined in Table 1.

**Table 1:** Definition of complications in patients with chronic post-sternotomy wounds undergoing pedicle flap reconstruction. NPWT: Negative pressure wound therapy

	Complication	Definition
Flap related	Bleeding	Direct postoperative bleeding requiring operative control
	Hematoma	Blood collection around the flap requiring intervention
	Marginal necrosis	Wound dehiscence or minor flap necrosis, mostly in the inferior part (distal end), requiring dressing changes for >3 weeks
	Moderate flap necrosis	Necrosis < 50% of the flap requiring debridement ± NPWT ± re-reconstruction either with skin grafting or flap reconstruction
	Flap loss >50% (severe flap necrosis)	Necrosis of more of 50% of the flap, requiring debridement ± NPWT ± re-reconstruction either with skin grafting or flap reconstruction
Donor-site related	Seroma	Fluid collection requiring intervention (drainage, aspiration)
	Necrosis	Any tissue necrosis resulting in skin defect, requiring debridement ± NPWT ± reconstruction with skin grafting

### Surgical technique

Latissimus dorsi and unilateral or bilateral pectoralis pedicle flap are the standard procedures for post-sternotomy wounds in our department. The final decision depended on surgeon’s preference, although the pectoralis flap was generally avoided for wide (i.e. > 10 cm) and deep defects. Each wound was firstly debrided and a negative pressure wound therapy (NPWT) with Vacuum-Assisted Closure (VAC) was applied. The system consists of a polyourethane foam sponge dressing, fitted in the wound and successively covered with a transparent adhesive drape. An evacuation tube was then inserted into the foam and connected with a vacuum pump under continuous controlled pressure suction at 100-120 mmHg. The foam was changed in regular time intervals under sterile conditions. Antibiotics were tailored specifically to each wound infection and the wounds were successively debrided and further treated with NPWT until healthy granulation tissue was achieved.

For the unilateral pectoralis flap, the major pectoralis muscle is elevated off its sternocostal origin and then epifascially advanced above the pectoralis minor muscle. The muscles are then sutured medially to each other. Large defects require bilateral elevation of the major pectoralis muscles, which is then advanced medially and sutured to each other without tension. The skin is then closed over two Redon-drains in double layer fashion. Skin grafting may also be required.

The latissimus dorsi flap can be raised as a myocutaneous or a muscle flap. The homonymous muscle is elevated after identification and preparation of the thoracodorsal vessels via an anterolateral approach. It is then tunneled under the skin and passed into the sternum pocket to fill the defect. The donor-site defect is closed over two Redon-drains in double layer fashion. The patient is then placed in the supine position and the flap is sutured in the new position. Muscle flaps require skin grafting.

### Statistical analysis

Normal distribution was determined using histogram plots, box plots and the Shapiro–Wilk test. Continuous data with a normal distribution are presented in mean-deviation form. Non-normal distributed variables are presented with medians and ranges. Categorical variables were compared using the two-sided Fischer’s exact test. We employed the two-tailed student t-test to compare continuous variables. A p-value of less than 0.05 was considered statistically significant. Data analyses were performed using SPSS version 17.0.

## Results

One-hundred one patients underwent pedicle flap chest wall reconstruction in our department over the study time period. Forty-nine of these were referred to us because of infected median sternotomy wounds after cardiac surgery. One patient did not meet the inclusion criteria (BMI < 18.5 kg/m^2^) and was excluded from the study. The total sample consisted of 48 patients, 24 males (50%) and 24 females (50%).

Histogram plots, box plots and Shapiro-Wilk test demonstrated a normal distribution appearance for all continuous variables except for the time to referral, the duration of NPWT therapy and the transfusion requirement. Overall mean BMI was 28.4 (6.1) kg/m^2^. Mean age was 67 (12) years. The younger patient was 35 years old and the older 86. The study group consisted of 28 patients with BMI > 25 kg/m^2^, who were compared with 20 normal-weight patients (control group). Overall time from sternotomy to referral was 48 days (range 11–5508).

### Demographic parameters

No demographic differences were observed between the two groups. The mean (SD) age of patients in the study group was 65 years (12) vs. 70 (12) in the control group (p = 0.14). The normal-weight group consisted of 8 women (40%) and 12 men (60%) and the overweight/obese group consisted of 16 women (57%) and 12 men (43%, p = 0.38). As expected, BMI was significant different between two groups. NW patients had a mean (SD) BMI of 22.7 (1.55) kg/m^2^, while OW/OB patients a mean BMI of 32.5 (4.75) kg/m^2^, p < 0.05). The study group contained less patients with ASA score 4 (7 out of 28, 25%) in comparison to the study group (8 out of 20, 40%), the difference not being statisticallly significant (p = 0.16). Nine NW patients (45%) had a NYHA score of 3–4 and eleven (55%) a NYHA score of 1-–. In the study group, the majority (57%) had a NYHA score of 3–4 (p = 0.56).

### Wound-related parameters

All wounds were cultured and 46 out of 48 (96%) grew pathogens. 20 (43%) contained more than one microorganism. In 9 cases (19.5%) the culture revealed an unspecific polymicrobial skin microflora, not retrospectively identifiable. The most common pathogen was Staphylococcus epidermidis (40%), followed by Enterococcus faecalis (20%), Pseudomonas aeruginosa (9%) and methicillin-resistant Staphylococcus aureus (7%).

Median time of referral to plastic surgery post-sternotomy was 48 days (range 10–5508 days) without significant differences between the two groups (47.5 vs. 49 days). The median duration of NPWT before flap reconstruction was also identical in both groups (21 days). The overall median NPWT duration was 20 days (1–49). One third of patients underwent complete sternectomy before flap reconstruction in both groups (p = 1).

All demographic and clinical preoperative features of the cases included are summarized in Table 2.

**Table 2:** Demographic and clinical characteristics of NW and OW patients requiring flap reconstruction after sternotomy

Preoperative parameter	Study group (n = 28)	Control group (n = 20)	p-value
Age	64.6 (11.9)	69.7 (11.5)	0.14
Sex: Male	12 (43%)	12 (60%)	0.38
BMI (kg/m^2^)	32.5 (4.75)	22.7 (1.55)	**< 0.05**
ASA-4 score	7 (25%)	8 (40%)	0.16
NYHA 3-4 score	16 (57%)	9 (45%)	0.56
Referral time after sternotomy (days)	48 (11–393)	49 (10–5508)	0.1
NPWT duration (days)	20.5 (1–39)	20.5 (0–49)	0.76
Complete sternectomy	10 (36%)	7 (35%)	1

The BMI difference was statistically significant,* p* was < 0.05

Time from sternotomy to referral is expressed in median range form, as it is not normally distributed. All continuous variables are normally distributed and therefore expressed in mean-deviation form. Categorical variables are expressed in terms of absolute (n) and relative (%) frequencies. Statistical significance: p-value<0.05

NS, not significant

### Perioperative parameters

There was a significant difference amongst both groups regarding duration of surgery (mean: 174 vs. 120. minutes, p < 0.05), in favor of the control group, because of patients undergoing pectoralis reconstruction (85 vs. 139 min, p < 0.05). Patients undergoing latissimus dorsi reconstruction did not differ in terms of duration of surgery in the two groups [mean (SD) time 205 (50) minutes, vs. 185 (48) minutes, p = 0.38]. Overall mean operation time was 152 min. Both groups showed equal transfusion rates. Blood loss had to be substituted with a median of 5 red cell concentrates (range 0-17) in the study group vs. 3 (range 0-14) in the control group, p = 0.25.

Overall, equal number of patients (n = 21, 44%) underwent latissimus dorsi and bilateral pectoralis major flap reconstruction. The unilateral form of the pectoralis flap was used in 6 cases (12%). In the study group the latissimus dorsi flap was most often applied (n = 15, 53%) followed by bilateral pectoralis flap (n = 10, 36%) and unilateral pectoralis flap (n = 3, 11%). On the contrary, the bilateral pectoralis flap was the most popular in the control group (n = 11, 55%) followed by latissimus dorsi flap (30%) and unilateral pectoralis flap (15%). The most important intraoperative parameters are presented in Table 3.

**Table 3:** Perioperative findings in mean (deviation) form

Outcome	Study group (n = 28)	Control group (n = 20)	p-value
OP length (min)	174 (59)	120 (59)	< 0.05
Reconstruction with latissimus dorsi flap (min)	205 (50)	185 (48)	0.38
Reconstruction with pectoralis flap (min)	139 (48)	85 (40)	< 0.05
RBC (units)	5 (0–17)	3 (0–14)	0.26
Flap selection			
Latissimus dorsi flap	15 (53%)	6 (30%)	0.14
Bilateral pectoralis flap	10 (36%)	11 (55%)	0.24
Unilateral pectoralis flap	3 (11%)	3 (15%)	0.68

Transfusion requirement in RBCs is presented in median range form, as it is not normally distributed. Categorical variables (flap selection) are expressed in terms of absolute (n) and relative (%) frequencies. Statistical significance: p < 0.05

NS, not significant

### Postoperative outcomes

The length of overall hospital stay (from admission to discharge) was equal in both groups. NW patients were discharged after a mean of 52 days, while OW/OB patients were hospitalized one week longer (59 days, p = 0.44).

Donor-site complications requiring intervention were observed in 30% of patients in both groups. One patient from each group developed a skin defect requiring skin grafting in the donor-site (p = 1). Seromas were equally frequent in both groups.

Flap-related complications were recorded in 16 patients (57%) of the study group and 7 patients of the control group (35%, p = 0.15). These included bleeding, hematoma or/and abscess formation as well as wound dehiscence ranging from marginal necrosis to flap loss > 50%. Marginal necrosis was equally frequent in both groups. Flap loss > 50% was observed in 8 (40%) and 2 (10%) in study and control subjects respectively (p = 0.17). The difference did not reach statistical significance, probably because of the sample size. The flap loss in the study group was observed mostly after bilateral pectoralis flap (n = 4) and latissimus dorsi flap (n = 3). The remaining defects were then closed with a latissimus dorsi flap (n = 3) or omentum flap (n = 3). If the initial defect was reconstructed with a latissimus dorsi flap, then the homonymous muscle from the other side was used. Two defects were covered with skin graft after an individually tailored VAC therapy.

Thirty-day mortality was similar in the two groups. In the study group, one female obese patient (BMI=32) developed an external iliac artery occlusion and despite immediate intervention, died on acute renal failure on the 28th day after reconstruction. In the control group three deaths, all in males, were observed. Two patients died on respiratory failure and one patient developed a gangrenous colitis and died of sepsis.

The postoperative outcomes of both groups are summarized in Table 4.

**Table 4:** Postoperative outcomes expressed in terms of absolute (n) and relative (%) frequencies

Outcome		Study Group (n = 28)	Control Group (n = 20)	p-value
LOS (days)		59 (30)	52 (37)	0.44
30-day-mortality		1 (3.5%)	3 (15%)	0.29
Flap-related complications	Bleeding	0	1 (5%)	0.42
	Hematoma	1 (3.5%)	1 (5%)	1
	Marginal necrosis	4 (14%)	3 (15%)	1
	Moderate flap necrosis	2 (7%)	0	0.5
	Flap loss >50% (severe flap necrosis)	8 (29%)	2 (10%)	0.16
	Total	16 (57%)	7 (35%)	0.15
Donor Site related complications	Seroma (latissimus dorsi cases)	6/15 (40%)	2/6 (33%)	1
	Wound dehiscence	1 (3.5%)	3 (15%)	0.3
	Necrosis	1 (4%)	1 (5%)	1
	Total	8 (29%)	6 (30%)	1

Statistical significance: p-value<0.05. NS= not significant

### Algorithm (Fig. 1)

**Fig. 1:**
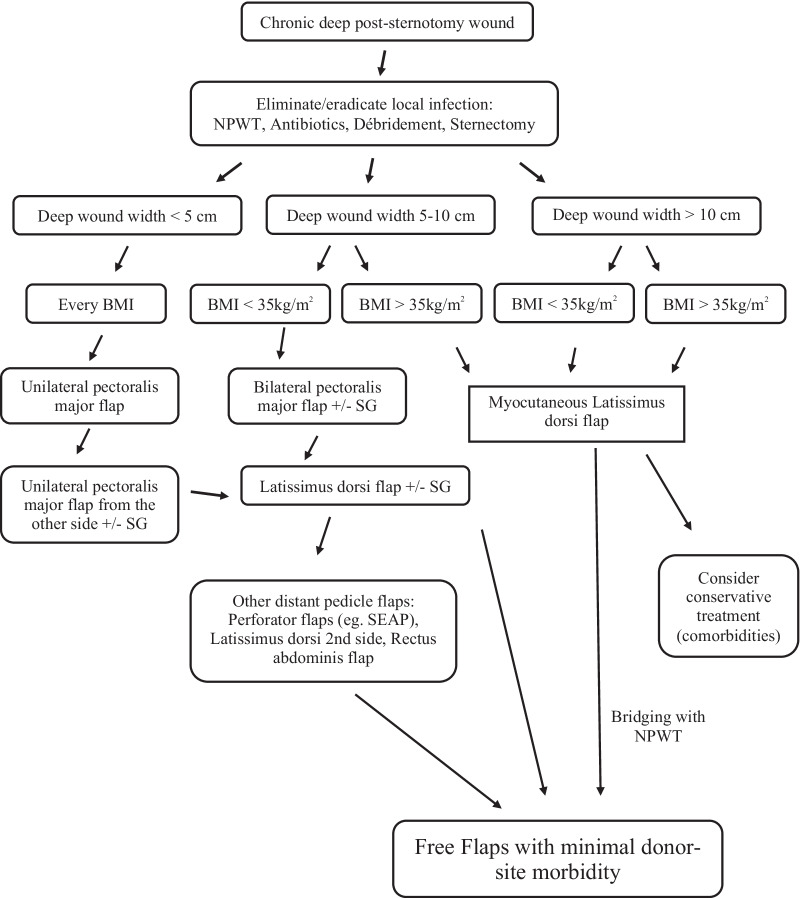
Proposed algorithm for chronic post-sternotomy wounds based on the patient’s BMI and the deep wound width. ΒΜΙ: Body Mass Index, SG: skin graft, NPWT: negative pressure wound therapy

Based on the above findings, we propose a management algorithm for deep chronic post-sternotomy wounds based on patient’s BMI and wound width (Fig. 1). For BMI, we set the cut-off value at 35 kg/m^2^, as above this value we observed most complications, not only because of the obesity, but also because these patients had severe comorbidities. Prior to reconstruction, local infection should be eradicated or at least eliminated, by means of NPTW, antibiotics and surgical débridement. Total sternectomy is often required (approximately one third of the cases). The resulting defect is classified according to its width, as small (< 5 cm), moderate (5-10 cm) and large (> 10 cm).

*Small* defects (i.e. width < 5 cm) can be covered with a unilateral pectoralis major flap irrespective of BMI. Mobilising the skin is usually sufficient for tension-less wound closure. The flap can, therefore, be raised as a muscle flap. Otherwise it can be combined with skin grafting. If the flap fails (i.e. flap necrosis > 50%), the same flap from the other side can be considered. If it also fails, the reconstruction can proceed with a latissimus dorsi flap. More flaps are rarely necessary.

*Moderate* defects (i.e. width 5–10 cm) in patients with BMI < 35 kg/m^2^ can be covered with a bilateral pectoralis major flap and a skin graft. In case of complications, the reconstruction algorithm proceeds with the latissimus dorsi flap. If it also fails, we recommend other distant solutions. These can be perforator flaps, e.g. the superior epigastric artery perforator flap, the latissimus dorsi flap from the other side or the rectus abdominis flap. However, given the high donor site morbidity, free flaps may also be directly considered.

Patients with *moderate defects and severe obesity* (BMI > 35 kg/m^2^) are not good candidates for bilateral pectoralis major flap, as it is associated with many complications. Even with adequate mobilization of the muscle and clinically tension-free wound closure, the weight of the breast in women in the supine position results in multi-layer wound dehiscence. In our experience, in such patients, skin graft wound healing is almost never uneventful. For these reasons we propose a myocutaneous latissimus dorsi flap, which is considered a technically easy and very reliable flap. If this flap fails, we recommend avoiding other distant pedicle flaps, as they are technically much more demanding so the possibility of increasing local morbidity is high. Conservative treatment (secondary intention healing) should be very carefully considered, because of medical comorbidities. If it is out of the question, free flaps with minimal donor-site morbidity should be considered after a bridging-therapy with NPWT for 7–10 days. We propose the same algorithm for patients with large (i.e. > 10 cm) defects.

## Discussion

Midline sternotomy has become the standard incision in cardiac surgery for open heart operations. Sternum wound infection often complicates sternotomies, resulting in substantial increases in mortality, morbidity and healthcare costs. A substantial number of such patients, previously treated with debridement and open packing, require muscle or omental flap reconstruction. The most popular myocutaneous flaps used for this purpose are the latissimus dorsi flap, the pectoralis flap and the rectus abdominis flap. Pedicle flap sternum reconstruction in overweight and obese patients can be challenging, because of technical difficulties and the increased risk of developing complications. To the best of our knowledge this is the largest series presenting early postoperative outcomes of pedicle flap reconstruction among different weight groups of patients with deep post-sternotomy wounds and the first to propose a BMI-based management algorithm for these patients.

The male gender has been identified as a predictor of sternal wound infections in cardiac surgical patients [9]. Our overall sample was balanced in terms of gender (50–50%), although a slight male predominance seems consistent in the existing literature [10]. This seems logical, as men are more often affected by and therefore operated upon heart disease. However, Tyson et al. reported a higher proportion of women (60%) in a population of 128 extreme obese patients (BMI > 45 kg/m^2^) undergoing cardiac surgery [11]. We also observed a female predominance in the OW/OB group, but, probably due to the small sample size, the differences did not reach statistical significance. The most common pathogens found in post sternotomy wounds, include Staphylococcus epidermidis und Enterococcus faecalis as in our study [12]. Average time of referral to plastic surgery post coronary artery bypass graft was 32 days in a UK series of 27 patients, while our patients were referred after a mean time of 48 days post-sternotomy [13].

Jones et al. favor the pectoralis major flap as first-choice treatment, followed by rectus abdominis, omentum flap and lastly the latissimus dorsi [10]. Lee et al. also suggest that upper sternal wounds be covered with the pectoralis major flap, however, they advocate the use of latissimus dorsi flap with fasciocutaneous extension as first choice treatment for lower sternum defects, less than 5 cm in width [2]. On the contrary, Weinand et al. preserve this flap for wounds exceeding 12 cm in width, recommending unilateral or bilateral pectoralis flap reconstruction for defects narrower as 6 and 12 cm respectively [8]. Hever et al., recommends superior sternum reconstruction with a turnover flap based on perforators of the internal mammary artery or bilateral pectoral advancement flap or the latissimus dorsi flap, whereas he recommends the rectus abdominis flap for inferior sternum reconstruction [14]. Greig et al. propose the use of either pectoralis major or combined pectoralis major and rectus abdominis flaps [13]. We find this approach more rational, although we set the thresholds at 5 and 10 cm. In our department, the latissimus dorsi flap is the second choice in small defects, preserved for cases where the pectoralis major flap fails. However, based on our experience, we recommend this flap as first-line reconstruction in patients with moderate defects and severe obesity as well as patients with large defects. In every case, the patient should be adequetely informed about the risks of all flaps before signing the informed consent [15].

NPWT has been proven to be a safe and effective modality in the management of post-sternotomy wounds, which shortens wound healing and hospital stay periods [16]. It is shown to increase granulation tissue formation through mechanical stresses exerted on the wound environment. NPWT can be applied as monotherapy, but most often is used as a bridge to surgery. It can also be used either as a primary treatment or after failure of a previous treatment. Gdalevitch et al. define VAC failure as death from sepsis, presence of clinically or/and radiologically confirmed 1-year infection recurrence or need for surgical closure, due to wound or patient deterioration. They identified several predictors of VAC failure, including wound depth ≥4 cm, positive blood cultures, high grade of bone exposure and sternal instability, the combination being the most important. They report a median NPWT duration of 28 days [17]. This is not inconsistent with our results (median 20 days), as we performed flap reconstruction and therefore used NPWT as a precursor to surgery [17]. Recent studies advocate short-term NPWT use and early reconstruction, after a mean time of NPWT of 11 days. Prolonged NPWT use is associated with sternal breakdowns, infection, osteomyelitis and erosion of the ascending aorta, caused from pathogen incorporation rather than mechanical damage [18]. We recommend NPWT for wound conditioning during the intervals between the flaps. When combined with repeated surgical debridements and antibiotics it can reduce wound contamination and achieve clean conditions. Moreover, early reconstruction can avoid the development of deep infected sternal wounds.

The mean duration of surgery was significantly longer in the OW/OB group in our study (174 vs. 120 min). Although one can attribute this difference to the larger proportion of latissimus dorsi flaps, performed in the study group (53% vs. 30%), this difference is, though, not statistically significant. Subgroup analysis revealed that it was pectoralis flap reconstruction that had a significant longer duration of surgery (139 vs. 85 min, p < 0.05). Duration of surgery among patients having latissimus dorsi flaps did not differ in the two groups. Therefore, this approximately 50-minute difference observed in our series, seems to indicate technical difficulties, during preparation of the pectoralis muscle, in the OW/OB group. This is one more argument in favor of latissimus dorsi flap in OW/OB patients with moderate defects.

We found an overall LOS of over 50 days. OW/OB patients were hospitalized one week longer. Gdalevitch report an overall LOS of 48 days [17]. Jones et al. managed to significantly reduce the postoperative hospital stay over the years at 12.4 days by increasing the use of home intravenous therapy and the use of single-stage debridement and closure [10].

Although obesity is clearly defined as an independent factor for developing post-sternotomy sternal wound infection, its impact on pedicle flap outcomes is not well documented. It is reported to influence surgical outcomes twofold: through a direct impact on surgical procedures due to its physican effects, and indirectly, as it predisposes the patient to higher comorbidity [19]. Despite this fact, we did not observe statistical differences in total complication rates. Much of the morbidity related to pedicle sternum flap reconstruction is attributed to sternal instability [20]. Local reasons (i.e. sternum overstretch) may also result in unreliable blood supply [21].

No statistically significant difference was observed in both BMI groups, although there were no deaths in the OW/OB group. The small sample size however, decreases the ability to recognize small differences if present. A larger sample size would here minimize any sampling error. Similar to our results, Jones et al. reported an overall mortality rate of 8,1%, in a series of 409 patients, defining mortality as death from any cause within 30 days of flap reconstruction or at any interval if the death was related to sternal wound infection. Given the critical condition of patients developing post-sternotomy wound dehiscence, a further mortality reduction seems not realistic [10]. The trend toward immediate coronary perfusion made the problem more severe as more critical patients requiring sternum reconstruction are now admitted [10]. In our series no death was directly related to sternal infection within 30-days of reconstruction.

Our study has several limitations, inherent to retrospective studies. Firstly, it is based on medical records, which are captured for clinical purposes and could have lacked precision compared with records prospectively captured for research purposes. Secondly, it is observational and has a sample size insufficient to permit an accurate statistical study of risk factors. Therefore, several differences observed did not reach statistical significance. We also did not distinguish between acute and chronic (>6 weeks) sternum infections [22]. Moreover, we underline the potential issue of heterogeneity in characteristics of the utilized pedicle flaps, which were not analyzed separately.

We conclude that wound width and BMI can aid the decision-making process for patients with infected sternal wounds after cardiac surgery requiring pedicle flap reconstruction. However, in our case series analysis, OB/OW patients were not found to be at increased risk for worse postoperative outcomes, but were associated with a longer duration of surgery. Further studies with larger sample sizes are required in order to elucidate a causal association between overweight/obesity and postoperative outcomes in this patient group.

## Data Availability

The datasets used and/or analysed during the current study are available from the corresponding author on reasonable request.

## Abbreviations

ASA: American society of anesthesiologists
BMI: Body mass index
LOS: Length of stay
NPWT: Negative pressure wound therapy
NW: Normal-weight patients
OW/OB: Overweight/obese patients
VAC: Vacuum-assisted closure
